# Probing short-range protein Brownian motion in the cytoplasm of living cells

**DOI:** 10.1038/ncomms6891

**Published:** 2014-12-23

**Authors:** Carmine Di Rienzo, Vincenzo Piazza, Enrico Gratton, Fabio Beltram, Francesco Cardarelli

**Affiliations:** 1 Center for Nanotechnology Innovation @NEST, Istituto Italiano di Tecnologia, Piazza San Silvestro 12, 56127 Pisa, Italy; 2 NEST, Scuola Normale Superiore and Istituto Nanoscienze-CNR, Piazza San Silvestro 12, 56127 Pisa, Italy; 3 Laboratory for Fluorescence Dynamics, Department of Biomedical Engineering, University of California, Irvine, California 92697, USA

## Abstract

The translational motion of molecules in cells deviates from what is observed in dilute solutions. Theoretical models provide explanations for this effect but with predictions that drastically depend on the nanoscale organization assumed for macromolecular crowding agents. A conclusive test of the nature of the translational motion in cells is missing owing to the lack of techniques capable of probing crowding with the required temporal and spatial resolution. Here we show that fluorescence-fluctuation analysis of raster scans at variable timescales can provide this information. By using green fluorescent proteins in cells, we measure protein motion at the unprecedented timescale of 1 μs, unveiling unobstructed Brownian motion from 25 to 100 nm, and partially suppressed diffusion above 100 nm. Furthermore, experiments on model systems attribute this effect to the presence of relatively immobile structures rather than to diffusing crowding agents. We discuss the implications of these results for intracellular processes.

## Abstract (web summary)

Models for protein diffusion in cells assume a large macromolecular crowding effect. Here Di Rienzo *et al.* visualize GFP diffusion at the millisecond timescale to observe unobstructed Brownian motion in mammalian cells for distances up to 100 nm, revealing minimal influence of macromolecular crowding.

## Graphical abstract

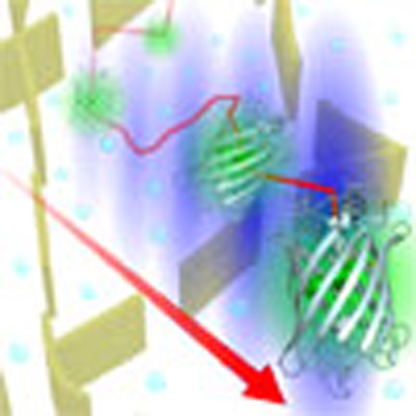

Since the pioneering works by Einstein and von Smoluchowski^1^,^2^, it is universally accepted that transport of mesoscopic particles in simple solvents is governed by Brownian diffusion. It is also recognized that this paradigm dramatically fails to describe the motion of molecules in complex biological media, such as the interior of cells^3^,^4^. Three decades of biophysical investigations have characterized a number of ‘anomalous’ phenomena associated with the translational motion of molecules in cells, the most common being sub-linear, power-law increase of the mean square displacement (MSD) as a function of time, strongly suppressed and time-dependent diffusion coefficients, heterogeneous diffusion and a fraction of immobile particles^3^. Different models and theoretical approaches have been pursued that generically account for anomalous phenomena. A class of models is represented by the continuous-time random walks^5^: here particles spend most of the time bound to a ‘trap’ with a probability to unbind that depends on the depth of the trap. Anomalous transport can be generated by assuming binding time distributions such that the mean-binding time becomes infinite. Although trapping may appear rather natural, because of the chemical interactions of molecules in the cell^6^, the same considerations cannot apply to the case of molecules with negligible or no interactions with the environment. Anomalous phenomena are deemed possible even in the absence of binding within a different class of models (for example, the fractional Brownian model^7^ and ‘Langevin equations’-based models^8^) which point to the viscoelastic properties of the medium. In these descriptions, the intracellular medium is primarily considered a homogeneous dispersion of biological polymers (for example, microtubules, actin filaments, DNA, RNA) and layers (for example, membranes). Although the viscoelastic response well predicts the behaviour of microscopic molecules (that is, molecules of a size comparable to intracellular components)^9^, the observation of suppressed translational diffusion even in the case of nanoscopic inert molecules (for example, green fluorescent protein (GFP)) is somewhat surprising, especially in light of the aqueous molecular environment measured on the spatiotemporal scale of rotational diffusion^10^,^11^. In this regard, a last class of models (also known as ‘Lorentz’ models^12^) contemplates the case in which the intracellular medium cannot be considered a homogeneous environment for the diffusing molecule, that is, the tracer is a *nano*sized, inert molecule such as a small solute or globular protein. Here the tracer explores a heterogeneous space which acts as a system of obstacles to diffusion, and in turn is responsible of anomalous dynamics^13^. Such obstacles can be readily identified with intracellular macromolecular crowding agents, that is, the high concentration of solutes, and densely packed, heterogeneous structures^9^,^14^. However, different descriptions of crowding are possible, which can be ideally divided into two main classes, according to the spatial distribution of the crowding agents assumed: homogeneously distributed and diffusing^15^,^16^, or spatially organized into relatively immobile structures^13^,^17^. Both views received support from selected *in cuvette* measurements^18^,^19^. Homogeneously distributed and diffusing crowding agents are expected to suppress translational motion even at the molecular scale (nanoscale), however, whereas heterogeneous structures would admit unobstructed translational motion as in dilute solutions up to the characteristic spatial scale of macromolecular crowding organization, where suppressed motion will occur. Unfortunately, these descriptions are indistinguishable on the basis of existing studies in live cells owing to the lack of techniques capable of probing crowding with the required temporal and spatial resolution. It is thus clear that the possibility to experimentally compare molecular displacements at very short timescales in cells with those in dilute solutions would provide a signature discriminating the different theories and provide insight into the physical origin of anomalous phenomena. Here we tackle this issue by the implementation of a novel image correlation spectroscopy analysis that can probe the nanoscale displacements of molecules in the three-dimensional (3D) cell interior with no *a priori* assumptions on their diffusion properties. We start from multiple scan speed image correlation spectroscopy of fluorescence fluctuations, previously presented by Gröner *et al.*^20^ and used to build spatially resolved diffusion maps in cells. Here, however, rather than using the different pixel dwell/line scan times to generate a temporal autocorrelation function for every pixel shift, we extract the characteristic molecular MSD for each scan speed (hereafter named *i*MSD). In other words, each scan speed is used as a filter to select the characteristic temporal scale of molecular displacements that significantly contributes to the measured correlation function (Fig. 1). This approach covers a hitherto unexplored dynamic range, as determined by available pixel dwell times, from 0.5 μs to several milliseconds, and gives selective access to average molecular displacements much smaller than the diffraction limit. Thanks to these two properties, a direct comparison between protein nanoscopic translational dynamics in dilute solutions and in live cells is made possible also for the case of very fast dynamics. This method is applied to the study of the dynamics of a paradigmatic inert, nanoscopic molecule (GFP) in the cytoplasm of living cells as compared with dilute solutions. Thanks to this comparison, we prove short-range, unobstructed, Brownian protein translational motion within the cytoplasm at the temporal scale from 10^−6^ to ~2 × 10^−5^ s, corresponding to protein mean displacements from 25 to 100 nm. Above 100 nm, a clear deviation from the Brownian behaviour typical of dilute solution is observed, thus matching what reported in the literature thus far. Experiments on model *in cuvette* systems do confirm that the observed phenomenology can be explained as the result of excluded volume effects because of spatially organized intracellular structures rather than to diffusing crowding agents. We believe that these findings represent a breakthrough in the field, and a paradigm shift of the general concept of intracellular diffusion at the nanoscale. We expect that the experimental observation of protein short-range Brownian motion in the interior of cells could revolutionize the way biochemical reactions are modelled, with impact on our understanding of virtually all intracellular processes.

## Results

### From RICS at tunable timescales to *i*MSD

**Figure 1:**
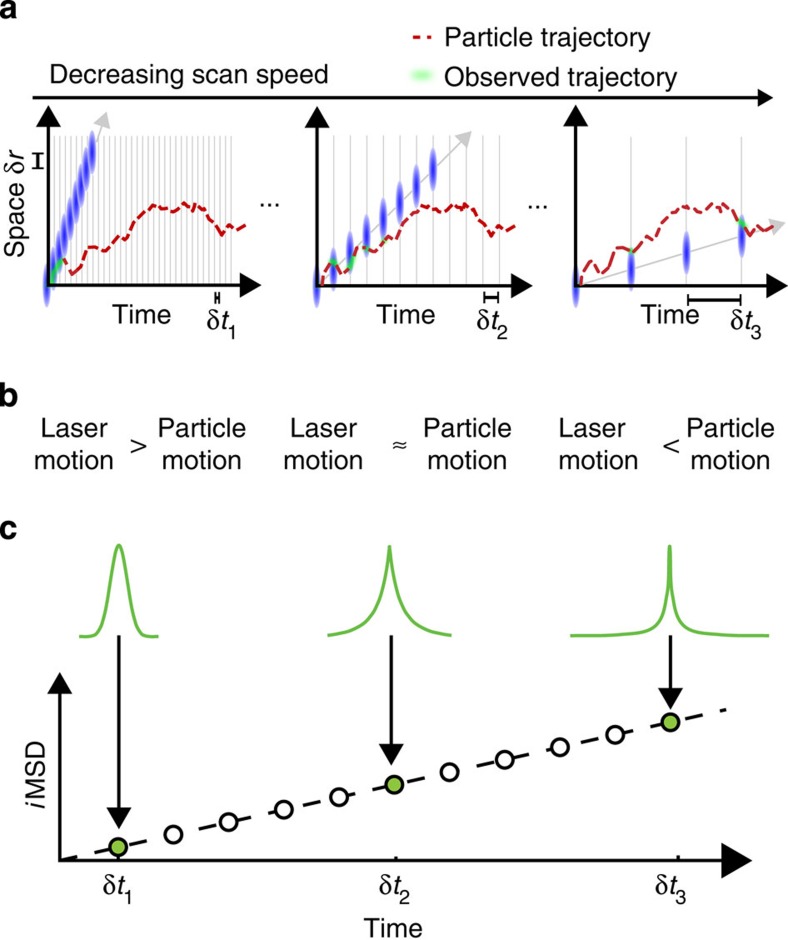
*i*MSD analysis of molecular motion in 3D: GFP Brownian diffusion in dilute solution. (**a**) Pictured experiment: ‘fluorescent’ molecules freely diffuse distributing in space and time. Scanning the samples with decreasing speed allows measuring the average particle displacements over a wide spatiotemporal scale. (**b**) When the scanning is faster than the particle dynamics, the particle image corresponds to the autoconvolution of the instrumental point spread function, which is well approximated by a Gaussian profile. On the other hand, when scanning speed decreases, the particle starts to move significantly, that is, the correlation function squeezes in space becoming much larger than the PSF. Equation 15 in Supplementary Information describes this deformation effect as a function of the particle average displacement, and allows recovering the *i*MSD as a function of time delay. (**c**) Schematic representation of the *i*MSD for a molecule diffusing in 3D.

**Figure 2:**
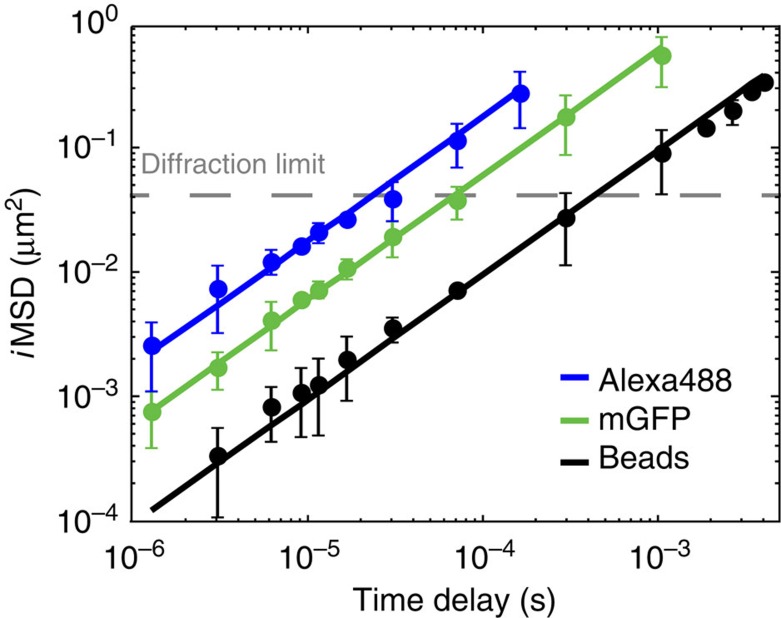
*i*MSD in dilute solution. Experimental *i*MSD values at the different timescales for differently sized molecules in dilute solution at 37 °C. Monomeric GFP (*N*=7 measurements, green dots) shows a linear behaviour in time, as expected for Brownian motion: fit by a free diffusion model (equation 13 in Supplementary Information) yields *D*_w_=134±4 μm^2^ s^−1^ (
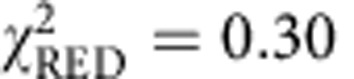
, *H*_r_=2.5 nm); Alexa488 (*D*_w_=428±15 μm^2^ s^−1^, *H*_r_=0.75 nm, 
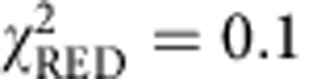
, *N*=7 measurements; blue dots) and 30-nm-diameter fluorescent beads (*D*_w_=22±0.5 μm^2^ s^−1^, 
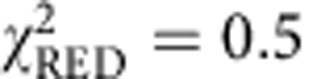
, *H*_r_=15 nm, *N*=7 measurements; black dots) are acquired under the same conditions. Data are mean values±s.d.

Technically, we perform consecutive raster image correlation spectroscopy (RICS)^21^ measurements, in which the pixel dwell time is progressively increased (that is, from 0.5 to 100 μs as described in Methods). Thus, we segment the temporal scale in such a way that the observed portion of particle trajectory along the scan direction varies according to scan speed (Fig. 1a). In particular, at high scan speeds only short-range particle displacements can be detected (Fig. 1a, left panel). Then, by decreasing the scan speed, progressively larger displacements are observed (Fig. 1a, middle-right panels). As a consequence, at high scan speeds, the particle image is only slightly deformed by the scan process, that is, it almost coincides with the autocorrelation of the instrumental point spread function (PSF; Fig. 1b, left). On the contrary, for decreasing scanning speeds, the apparent particle image is deformed because of molecular movement (Fig. 1b, middle-right), that is, the spatial correlation function becomes much larger than the PSF. We used a Gaussian interpolation of experimental correlation functions as an algorithmic way to obtain an estimate of the width of the particle-displacement distribution for each scan speed. This is quantitatively described by equation 15 in Supplementary Information and allows the recovery of the particle MSD directly from the raster scan images (*i*MSD, Fig. 1c). Notably, this approach covers a hitherto unexplored dynamic range, from 0.5 μs to several milliseconds (Supplementary Fig. 1). We used untagged monomeric GFP as an example of fluorescent tracer with no specific interactions with the environment, and suitable for both in cell^22^ and in *cuvette*^10^ experiments. As a preliminary experiment, we performed measurement on GFP recombinant proteins in dilute solutions (Fig. 2, green line). The measured average GFP displacements are reported against the corresponding timescale of observation on a double-logarithmic representation, as a means to easily identify the linear dependence typical of Brownian motion and possible deviations from it. As reported in the plot in Fig. 2, the *i*MSD values show the expected linear behaviour in time, clear indication of GFP Brownian motion in the dilute solution over the entire spatiotemporal scale observed. This outcome allowed us to obtain a diffusion coefficient (*D*_w_) of 134±4 μm^2 ^s^−1^ at 37 °C (equation 13 in Supplementary Information), corresponding to the expected hydrodynamic radius (*H*_r_) of the tracer (2.5 nm), in keeping with previous experimental estimates^23^,^24^,^25^,^26^. As expected, the experimental distributions of molecular displacements in dilute solution are well described by the Gaussian algorithm used for data interpolation (Supplementary Fig. 2). This accordance has been also verified by simulating the 3D diffusion of a point-like particle in solution (Supplementary Fig. 3). The capability to reveal the Brownian behaviour in dilute solution was further assessed with very differently sized molecules. In particular, the approach presented in this paper is suitable for tracking molecules with a wide range of diffusion coefficients (for example, from 500 μm^2^ s^−1^ typical of a small organic dye to 20 μm^2^ s^−1^ of a 30-nm particle; Fig. 2, black solid and dashed lines, respectively). It is also worth noting that average displacements down to 20–30 nm can be measured, thus demonstrating the ability of this approach to resolve molecular displacements well below the diffraction limit (dashed grey line in Fig. 2).

### *i*MSD analysis of GFP motion in the cell cytoplasm

**Figure 3:**
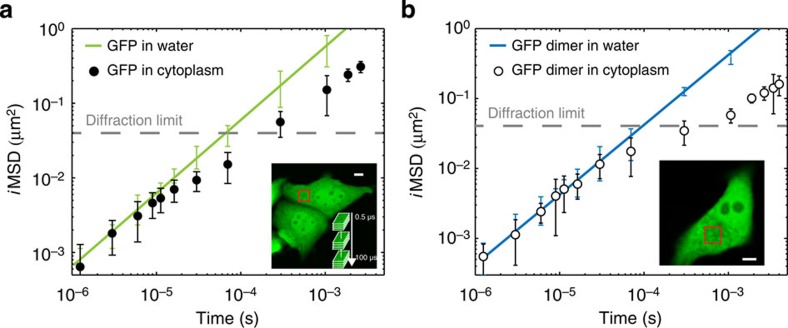
*i*MSD analysis of GFP diffusing in the cell cytoplasm. (**a**) GFP is transiently transfected into living CHO cells and analysed under physiological conditions. Arbitrary micrometre-sized areas in the cell cytoplasm are imaged sequentially at tunable timescales (inset). The *i*MSD values at the different timescales in the cytoplasm are reported in a double-logarithmic representation, as average of *N*=3 experiments, *n*=24 cells (black dots) and compared with the *i*MSD values obtained in solution (dashed green line, taken from Fig. 2). GFP translational motion below 2 × 10^−5^ s (corresponding to displacements in the 25–100 nm range) matches that observed in solution, indication of Brownian motion. Thus, it can be well described by the diffusion equation (*χ*^2^=0.42, *P*<0.01). However, the latter description does not apply to *i*MSD values above 2 × 10^−5^ s (*χ*^2^>>25, *P*>0.995) where a strong suppression in the GFP translational mobility is detected. (**b**) *i*MSD of GFP dimer in cytoplasm: double-logarithmic representation as average of *N*=2 experiments, *n*=9 cells (black dots). *D*_0_ (95±5 μm^2^ s^−1^) and *D*_inf_ (12±3 μm^2^ s^−1^) are estimated by a linear fit of the *i*MSD below 5 × 10^−5^ s and above 1 × 10^−3^ s, respectively. The green bars represent the GFP dimer *i*MSD measured in cell lysate and the green line represents the linear fit corresponding to *D*_w_=105±4 μm^2^ s^−1^. Scale bar, 5 μm. Data are mean values±s.d.

**Figure 4:**
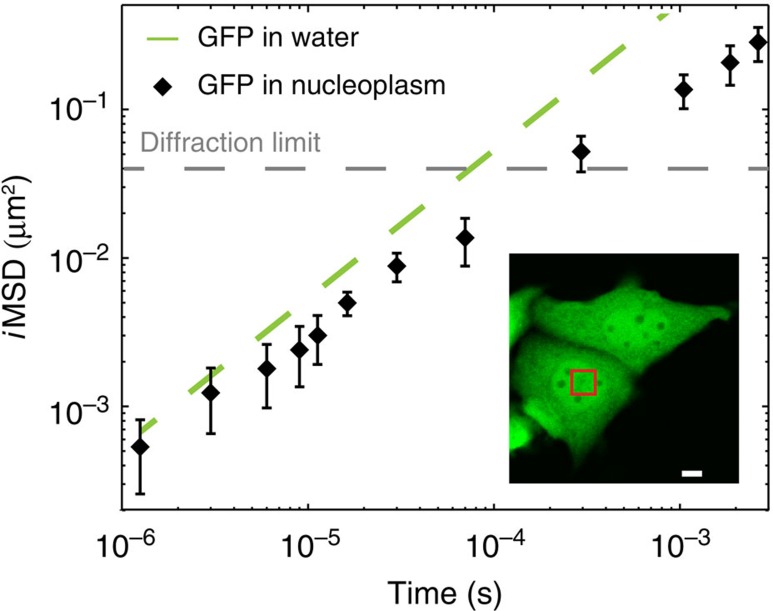
*i*MSD analysis of GFP diffusing in the cell nucleus. (**a**) GFP is transiently transfected into living CHO cells and analysed under physiological conditions (inset). Arbitrary micrometre-sized areas in the cell nucleus are imaged sequentially at tunable timescales. The *i*MSD values at the different timescales in the nucleoplasm are reported in a double-logarithmic representation, as average of *N*=3 experiments, *n*=22 cells (black squares) and compared with the *i*MSD in solution (dashed line, taken from Fig. 2). The GFP motion in this compartment is never coincident with that in dilute solution. Conversely, it is suppressed over the entire spatiotemporal scale observed. Also, no clear crossover spatial scale is visible over time. Scale bar, 5 μm. Data are mean values±s.d.

Taking the results in dilute solutions as a reference of Brownian dynamics, we investigated the motion of monomeric GFP transiently transfected into living CHO-K1 cells in physiological conditions (Fig. 3a, inset). We targeted arbitrary μm-sized areas in the cell cytoplasm and performed sequential raster scans at tunable timescales, as previously described. GFP average displacements at different timescales are reported in Fig. 3a (solid black dots, average of *N*=3 experiments, *n*=24 cells), and compared with those in solution (dashed green line, taken from Fig. 2). Remarkably, the hitherto unexplored timescale below 2 × 10^−5^ s reveals the unobstructed motion of GFP at the nanoscale in the cytoplasm of a living cell. In detail, *i*MSD values in the range from 25 to 100 nm match those measured in dilute solutions on the same timescale. This agreement suggests that 3D Brownian motion of an inert globular protein is possible on this spatiotemporal scale in the cell cytoplasm. In fact, the experimental distribution of GFP displacements on this spatiotemporal scale is well described by the Gaussian algorithm used for data interpolation (Supplementary Fig. 4). Consequently, fit to the free diffusion model (equation 13 in Supplementary Methods) for *t*<2 × 10^−5^ s yields an estimate of the GFP diffusion coefficient in the cell (*D*_0_) of 126±3 μm^2^ s^−1^ at 37 °C (*D*_0_/*D*_w_~1). This finding implies that cytoplasmic viscosity at this scale (hereafter referred to as nano-viscosity) is almost equal to that of dilute solutions, in agreement with what was concluded from local measurements of protein rotation by time-resolved fluorescence anisotropy^10^,^11^. By contrast, the *i*MSD values measured at timescales above 2 × 10^−5^ s show a clear deviation from the Brownian regime measured in a dilute solution (Fig. 3a). This deviation implies reduced protein translational motion, thus matching what is typically reported in the literature thus far on similar timescales^3^. As a further proof of such a deviation, the Gaussian algorithm used for data interpolation is not sufficient to satisfactorily describe the GFP experimental correlation function in this regime (Supplementary Fig. 4, for example, 20 μs pixel dwell time). However, it must be noted that MSD-based approaches in general are not sufficient to fully describe anomalous phenomena because of the lack of validity of the central-limit theorem^3^ (that is, other parameters in addition to ‘width’ are needed to describe the distribution of molecular displacements). Taking the diffraction limit as a spatial reference (dashed grey line in Fig. 3a), we can estimate that GFPs in about 200 μs displace slightly more than the diffraction limit spot size in the cytoplasm. This indicates an almost threefold suppressed (long-range) motion as compared with dilute solutions (~70 μs to travel the same distance), in keeping with several previous measurements on GFP or other globular proteins by several approaches^10^,^26^. This in turn confirms the usual observation of an apparent increased viscosity in the cytoplasm (hereafter referred to as micro-viscosity) compared with the dilute solution, but limits its significance to length scales larger than 100 nm. On this spatiotemporal scale, the *i*MSD of GFP returns to be approximately linear in time, and consequently well described by the Gaussian interpolation (Supplementary Fig. 4). Hypotonic conditions forcedly introduce water into cells and move the crossover between unobstructed and suppressed motion to 5 × 10^−5^ s (Supplementary Fig. 5). It is worth noting that the overall phenomenology observed here in the cell cytoplasm (double-linear representation of the *i*MSD versus time and double-logarithmic representation of *D*_app_ versus time are also reported in Supplementary Fig. 6) was already predicted *in silico* by Saxton for the diffusion of a particle in the presence of obstacles^27^ and experimentally described for lipids and proteins diffusing on the plasma membrane^28^,^29^. Notably, the characteristic crossover spatial scale between nano-viscosity and micro-viscosity at around 100 nm within the cell cytoplasm is also confirmed by using a GFP dimer (Fig. 3b). Such a spatial scale is almost coincident with the characteristic length of cytoskeleton-induced protein confinement measured on the plasma membrane (~150 nm for Transferrin Receptor^29^,^30^), and suggests a possible conserved spatial scale at which cells regulate dynamic processes. It is also worth noting that the observed phenomenology cannot be interpreted as the result of a mixture of two populations of molecules with distinct diffusion coefficients (for example, *D*_1_=*D*_0_ and *D*_2_=*D*_0_/3), as this latter case would have produced a linear *i*MSD over the complete temporal scale, as shown in Supplementary Fig. 7. It is worth noting that the *i*MSD values measured in a structurally different (for example, devoid of membranes and cytoskeleton) intracellular environment, such as the nucleoplasm, yield a different trend in which GFP motion is never coincident with that in a dilute solution, and no clear crossover timescale is visible throughout the considered spatiotemporal scale (Fig. 4a, see also double-linear representation of the *i*MSD versus time and double-logarithmic representation of *D*_app_ versus time in Supplementary Fig. 8). This in turn suggests that excluded volume effects on protein motion weight much less in the nucleoplasm than in the cytoplasm (see detail of the comparison between the two compartments at short times in Fig. 4b), in keeping with previous reports^31^.

### Validation of the protein motion model

**Figure 5:**
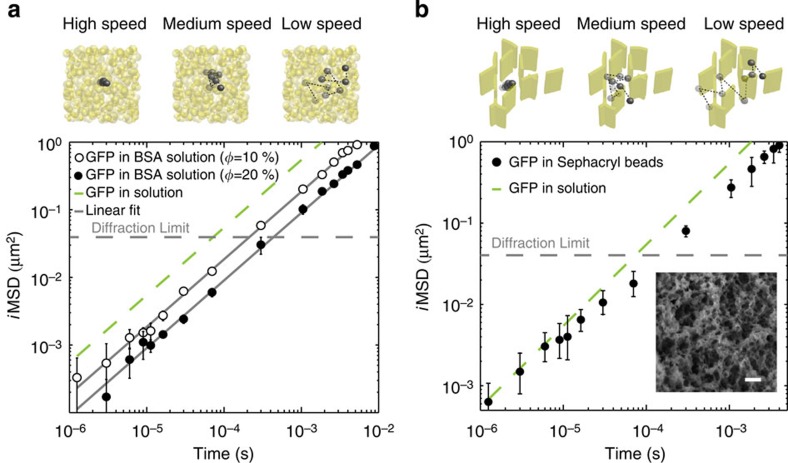
*In cuvette* validation of the protein motion model. (**a**) (Upper panel) Pictured GFP dynamics in the presence of colliding crowders. (**a**) (Lower panel) Experimental *i*MSD of GFP diffusing in BSA solution with different excluded volume (*ϕ*). For both tested excluded volume, the *i*MSD is not distinguishable from free diffusion and increasing excluded volume decrease molecular diffusivity (for *ϕ*=0.1, *D*=46±2 μm^2^ s^−1^ and *N*=7 measurements; for *ϕ*=0.2, *D*=24±2 μm^2^ s^−1^ and *N*=7 measurements). (**b**) (Upper panel) Pictured GFP dynamics in the presence of spatially organized obstacles. Fast scanning allows measuring GFP dynamics in the free space between obstacles. Decreasing scanning speed allows measuring the effect of boundaries on the GFP dynamics. (**b**) (Lower panel) The complete *i*MSD of GFP in Sephacryl beads is reported, as average of *N*=5 beads (black dots), and compared with the *i*MSD in solution outside the beads (dashed green line). The local dynamics (below 2 × 10^−5^ s) can be described well by GFP diffusion outside the Sephacryl beads (*χ*^2^=0.8, *P*<0.025). However, above 2 × 10^−5^ s, the latter is not representative for the whole *i*MSD (*χ*^2^>25, *P*>0.995) because of the reduction of long-range GFP mobility by the solid structure of the polymeric beads. The inset shows a representative scanning electron microscopy image of a Sephacryl beads, in which many submicrometer cavities are visible. Scale bar, 200 nm. Data are mean values±s.d.

Based on the knowledge acquired from the theoretical^13^,^15^,^16^,^17^ and *in cuvette*^18^,^19^ studies mentioned above, the observation of unobstructed Brownian-like motion in the cell cytoplasm prompts us to propose a model, in which the movement of inert molecules is mainly regulated by the excluded volume effect of immobile and/or slowly moving structures, rather than by freely diffusing solutes. To provide further verification of this hypothesis, we used our approach to measure GFP motion in two model systems that mimic the macromolecular crowding effects of homogeneously diffusing solutes and of excluded volume (Fig. 5). The former system was implemented with a crowded aqueous solution of bovine serum albumin (Fig. 5a). The retrieved *i*MSD values unequivocally depict GFP hindered motion over the entire spatiotemporal scale observed, with a mobility that decreases with increasing volume protein fraction (up to 20%) but does not depend on the timescale of the measurement. This result mirrors what was recently observed by quasielastic neutron backscattering measurements of protein self-diffusion in crowded solutions on a similar spatiotemporal scale^18^, and confirms the absence of distinguishable motion regimes in homogeneously crowded environments. Similar experiments conducted with a differently sized inert tracer (Alexa488) yielded analogous results (Supplementary Fig. 9a). For the excluded volume case, the model system studied consists of nanostructured polymeric beads (Fig. 5b). Here GFP molecules move in a heterogeneous environment where crowding is represented by the excluded volume effect imposed by the bead structural organization (Fig. 5b, inset). Notably, the retrieved *i*MSD values at varying temporal scales show GFP short-range unobstructed motion as in dilute solution (that is, Brownian), followed by suppressed motion at later times (Fig. 5b; similar results using Alexa488, Supplementary Fig. 9b), in analogy to what we observed in cells. In the polymeric beads samples, which are devoid of diffusing solutes, the observed phenomenology must be interpreted as the effect of immobile structures acting as barriers to molecular motion. In this regard, it is worth mentioning that Novak *et al.* demonstrated using simulations that cytoskeleton filaments are unlikely to constitute diffusion barriers sufficient to suppress diffusion of molecules in the cytoplasm (they would need to fill ~90% of space!), whereas, by contrast, internal membranes (for example, the endoplasmic reticulum sheets, mitochondria, vesicles, Golgi apparatus and so on) appear to be more likely candidates^17^. Consistently with this idea, selective disruption of the microtubules network by treatment with 10 μM Nocodazole did not significantly alter GFP behaviour in the cytoplasm (Supplementary Fig. 10), suggesting two additional considerations: (i) microtubules are not essential to generate GFP overall behaviour; (ii) the increase in soluble protein content in the cell cytoplasm due to microtubule depolymerization is not sufficient to affect GFP short-range motion. The overall interpretation of GFP diffusion in terms of obstructed motion in a heterogeneous environment was further verified by simulations (Supplementary Figs 11 and 12). In particular, we simulated free diffusion of a tracer in a 3D space filled with overlapping disk-shaped obstacles (which approximate the intracellular environment^17^). Remarkably, such obstacles are sufficient to generate a theoretical MSD (calculated from particle trajectories) that is analogous to that measured in cells (Supplementary Fig. 11a). Also, the accordance between the theoretical MSD and the *i*MSD calculated by the present approach is within the experimental error in these conditions (Supplementary Fig. 11b,c). Please note that the retrieved correlation functions, analogously to what observed in cells, are well fitted by equation 15 in Supplementary Information both for short- and long-range particle displacements (Supplementary Fig. 12 and Supplementary Methods). Conversely, deviation from the Gaussian approximation is detected at an intermediate scale, corresponding to the anomalous regime. Here the quantification of the shape of the distribution of particle displacements achievable through particle trajectories allowed us to demonstrate that under these conditions the central-limit theorem is not valid (Supplementary Fig. 13), in keeping with previous reports^13^.

## Discussion

Here we present an experimental approach that benefits from both the high temporal resolution of fluorescence-fluctuation spectroscopy and the nanometer accuracy typical of particle tracking. We were able to probe the molecular diffusion of an inert, nanoscopic protein probe (GFP) in the 3D intracellular environment down to the hitherto unexplored nanoscale, both in time and space. Using this approach, we unveiled the occurrence of GFP unobstructed Brownian translational motion in the cytoplasm at the temporal scale from 10^−6^ to ~2 × 10^−5^ s, corresponding to protein mean displacements from 25 to 100 nm. It is worth mentioning that a similar spatiotemporal scale has been investigated by correlation spectroscopy on 5-nm-radius fluorescently labelled gold beads in the cytoplasm and nucleus of live cells^32^. The authors demonstrate that the measured anomalous correlation function can be explained in terms of a viscoelastic response of the intracellular fluids. However, the data fitting to a viscoelastic model does not allow evaluating the occurrence of short-range molecular unobstructed motion, as done here where we have no *a priori* assumptions on the molecule diffusion properties. At this point, it is legitimate to ask whether the same level of information presented here may be accessed by different approaches previously described. Historically, fluorescence recovery after photobleaching, fluorescence correlation spectroscopy (FCS) and single-particle tracking have been used to address complementary aspects of 3D transport in live cells^3^ (see also Supplementary Fig. 1 and Supplementary Note 1). However, fluorescence recovery after photobleaching^24^,^33^ and classical single-point FCS^34^ measurements are spatially limited by the detection spot of the confocal microscope and therefore yield an average over the details of molecular diffusion below 200 nm in radial and 600 nm in axial distance. Single-particle tracking could in principle provide an answer below that limit but tracking small molecules in 3D is technically challenged because of their rapid diffusion^35^. Several variants of FCS have been proposed to overcome these limitations (see also Supplementary Information). For instance, it was demonstrated that molecular diffusion laws can be recovered by fluctuation analysis at various spatial scales by increasing the laser spot size^36^,^37^,^38^. Based on this approach, however, only inferences can be drawn about molecular dynamic behaviour below the diffraction limit. A promising new avenue was opened by spatially modulating the fluorescence emission with stimulated emission depletion (STED) methods^39^. This technology, in fact, allows the direct observation of biological phenomena with sub-diffraction spatial resolution and with the high temporal resolution needed to track molecules in a 3D environment. STED-based lateral resolutions of ~40 nm allowed probing the nanoscale spatiotemporal dynamics of lipids and proteins in live cell membranes^40^,^41^. Although theoretically attainable, however, effective STED-based nanoscopy of biomolecules in the 3D intracellular environment has yet to be tested by experiments. The intrinsic trade-off between spatial and temporal resolution suggests a careful consideration of the experimental conditions required: a focal volume ~100-times smaller than the confocal one would in principle require a considerably higher fluorophore concentration. The influence of this on the physiological regulation of intracellular dynamic processes shall be thoroughly evaluated in forthcoming studies. It is worth mentioning that an alternative strategy to extract the MSD from fluctuation analysis has been proposed by Shusterman *et al.*^42^ The authors derived an expression of the single-point FCS correlation function that is directly related to the molecular MSD. Based on this, the MSD of DNA polymers in a homogeneous solution have been successfully measured well below the diffraction limit. Yet, in a heterogeneous environment such as the intracellular medium, the molecular MSD is dependent on the particular position in space selected, as demonstrated by FCS-based diffusion maps^25^. In this particular case, spatial sampling is indispensable in addition to temporal sampling in order to understand if the diffusion properties measured locally are representative of the whole environment. Here, based on a straightforward implementation of fluorescence-fluctuation analysis at variable timescales, we were able to probe experimentally the average diffusion properties of protein-sized molecules in the 3D cellular environment with an unprecedented temporal resolution. We stress that the presented approach requires no assumption of an interpretative model. This approach, combined with the use of an inert tracer, made it possible to clarify that ‘nanoscale’ diffusion in the cell can be as effective as in dilute solution. On the other hand, ‘microscale’ diffusion is reduced, but apparently free. Overall, our observations unveiled the connection between intracellular nano- and micro-viscosities. Both of these are of importance, the first in relation to diffusion processes, the second in relation to the structural organization of the cell. In our opinion, the results reported here represent a paradigmatic shift in our description of protein intracellular diffusion, from the 3D reduced motion in a continuum fluid of soluble and colliding crowding agents to the unobstructed motion in a partitioned fluid of spatially organized, impermeable macromolecular structures, similar to what already demonstrated on the plasma membrane^28^. Let us speculate that such a structural organization of the cell cytoplasm may act as a supramolecular regulatory system, guiding protein motion through spatially defined paths, like those imparted to GFP by the chromatin structure in the nucleus^22^. In this context, the present method can also represent a standard for subdiffusive transport both in cells and *in cuvette*, able to cross-calibrate the different experimental and theoretical approaches to crowded environments, as recently claimed by Saxton^43^. A crucially important development is the use of the present method to probe cellular physiology at an unprecedented scale. Key processes of cell function like the kinetics of biochemical reactions, the dynamics of protein folding/unfolding, the effectiveness of target-search processes and intracellular signalling pathways will necessarily be regulated at the same spatiotemporal scale revealed here by an inert tracer, but they will be influenced by the specific interactions of their molecular components with the environment (preliminary data on a functional protein, namely GFP-tagged Importin-α, are reported in Supplementary Fig. 14). In conclusion, we believe the present findings coupled with use of genetically encoded fluorescent markers pave the way to novel studies of biomolecular processes in live cells at the physiologically relevant spatiotemporal scale.

*Note added in proof:* A paper by Baum, M. *et al.* Retrieving the intracellular topology from multi-scale protein mobility mapping in living cells. *Nat. Commun.* 5:4494 doi: 10.1038/ncomms5494 (2014) appeared after our paper was submitted. Using FCS above the diffraction limit, Baum et al. find that the cellular architecture has a role in regulating the diffusion of small molecules in the cytoplasm. Our conclusion is similar but is based on measurements in the sub-diffraction regime, at the scale of 50–100nm

## Methods

### Confocal imaging and FCS setup

Confocal fluorescence image series were acquired with an Olympus FluoView 1000-ASW-2.0 confocal laser scanning microscope (Olympus) using a × 60 (numerical aperture=1.2) planApo water-immersion objective. For excitation, the 488 nm line of an Ar laser was used. The fluorescence light for all the fluorophores used (GFP, Alexa488, 30-nm radius green latex nanoBeads) was measured between 500 and 600 nm with the internal detector in Pseudo Photon Counting mode. The diameter of the detection pinhole was set to the size of 1 Airy disk. The laser power in the sample was kept less than 2 kW cm^−2^ to avoid triplet state dynamics. The laser power was measured in front of the objective lens using a PM100A Power Meter Console (ThorLabs). All experiments were carried out at 37 °C and 5% CO_2_ using an incubation chamber enclosing the microscope stage and body. Sequential image series at 16 bits are collected at a fixed pixel size of 50 nm while varying the pixel dwell time (0.5, 2, 4, 8, 10, 12.5, 20, 40 and 100 μs per pixel). Typically, a region of interest of 256 × 256 pixels (pixel size of 50 nm) was selected. The sequential scanning process can be fully automated by using the Time Series Controller. A complete acquisition typically takes 10 min. No programmed delay between frames is used to reduce statistical error. Irregular curves resulting from major instabilities are identified by distortions of the FCS and/or intensity trace, and are excluded from further analysis.

### Data processing

The whole data analysis workflow was developed and run in Matlab (MathWorks) using the Image Processing Toolbox. After importing the image files as data matrices, the contribution to correlation due to immobile background and slowly moving intracellular structures was removed by applying the moving average filter, as described elsewhere^21^. The average correlation matrix is calculated according to equation 2 and fitted to equation 15 (Supplementary Information) in order to obtain the *i*MSD. We use this procedure as an algorithmic way to obtain an estimate of the width of the *i*MSD distribution for each scan speed. The whole process is repeated for every measured pixel dwell time in order to obtain the complete *i*MSD. All measurements in which the cell significantly moves or bright vesicles cross the ROI are discarded from analysis to avoid artefacts. The *i*MSD points shown in the figures were obtained by averaging couples of adjacent points to reduce the noise.

### Cell culture and treatments

CHO-K1 cells (CCL-61, ATCC) were grown in DMEM-F12 medium supplemented with 10 % (v/v) fetal bovine serum and maintained at 37 °C and 5% CO_2_. Plasmid encoding for EGFP (GFP elsewhere) and EGFP-EBFP (GFP dimer elsewhere) have been described in a previous work^44^. Cells were transiently transfected using Lipofectamine (Invitrogen) according to the manufacturer’s protocol. For swelling experiments, we prepared a hypotonic solution mixing 0.5 ml of normal cell medium with 0.5 ml of deionized water. Cell medium was replaced with hypotonic solution and cells were incubated 20 min before imaging. Cell lysate was obtained from transiently transfected CHO cells. In brief, cell were washed three times with PBS buffer at pH=7.4, detached with a cell scraper and suspended in 0.5 ml of PBS. After centrifugation at 9 × 10^3^*g* for 1 min, cells were suspended in 0.1 ml of PBS and sonicated for 30 min in a sonicator bath. After further centrifugation at 9 × 10^3^*g* for 15 min, the supernatant is collected. The viscosity of obtained solution was checked by measuring EGFP monomer diffusivity by RICS. The measured diffusivity of *D*=130±5 μm^2^ s^−1^ confirm a viscosity of the obtained solution close to that of water.

### Recombinant EGFP expression and purification

Expression of Strep-tagged EGFP was induced by IPTG into *Escherichia coli* strain BL21(DE3) (Invitrogen). Starter cultures were grown overnight in Luria-Bertani Medium (10 g of Bacto tryptone, 5 g of yeast extracts, 5 g NaCl and 1 ml of 1 M NaOH in 1 l of water) containing 100 mg ml^−1^ of ampicillin at 37 °C. The maximum yield was obtained at 30 °C 30 h after induction, as determined by SDS–polyacrylamide gel electrophoresis gel of the total lysate. Proteins were purified to homogeneity at 4 °C by an affinity chromatography step with a streptactin column (IBA), according to the manufacturer’s instructions.

### Preparation of dilute solutions

Alexa488 fluorophore (Sigma-Aldrich) was dissolved in water at a concentration of 100 nM. A 50-μl drop of this solution is directly put to the bottom glass of a Petri dish. In the case of monomeric GFP, a 50-μl drop of 100 nM solution is put to the bottom glass of a Petri dish previously treated for 1 h with a 1% BSA solution to prevent protein sticking to the glass surface. 10-μl solution of 30-nm radius green latex nanoBeads (Sigma-Aldrich) is dissolved in 90 μl of water and vortexed for 5 min. This solution is then filtered with 220-nm polyvinylidene difluoride syringe filter to remove particle aggregates and 900 μl more are pushed into the filter to recover the particles. 50 μl of the resulting solution are used for fluorescence microscopy. In all cases, the acquisition is performed at 5 μm above the Petri dish glass, at 37 °C. The concentration of monomeric EGFP stock solution was calculated from *A*_486_ using *ε*=61,000 cm^−1^ M^−1^.

### Preparation of Sephacryl beads solution

Sephacryl beads solution (S1000; GE Healthcare Lifesciences) was diluted (1:1,000) in a 100-nM GFP or Alexa488 water solution. Isolated beads can be found on the coverglass (pre-treated with a 1% BSA).

### Preparation of BSA solution

Following the protocol described by Roosen-Runge *et al.*^18^, lyophilized albumin from bovine serum (Sigma-Aldrich) is dissolved in polyphosphate buffer (PBS) in order to obtain a solution with an excluded volume (*ϕ*) 0.3 according to the relation:


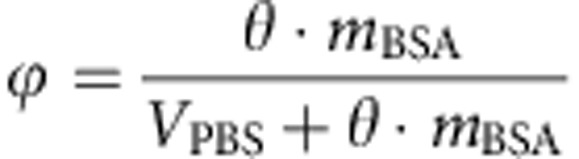


where *θ*=0.735 ml g^−1^ is for BSA, *m*_BSA_ is the mass of lyophilized BSA and *V*_PBS_ represents the volume of PBS solution. In order to gain the desired *ϕ*, this solution was diluted with the water solution of the fluorophore.

## Author contributions

C.D.R. designed the project, performed the experiments, analysed the data and wrote the paper. V.P. provided advice on experimental design and data evaluation. E.G. provided advice on experimental design, data evaluation and interpretation, wrote the paper. F.B. supervised the project, provided advice on experimental design, data evaluation and interpretation and wrote the paper. F.C. designed the research, analysed the data and wrote the paper.

## Additional information

**How to cite this article**: Di Rienzo, C. *et al.* Probing short-range protein Brownian motion in the cytoplasm of living cells. *Nat. Commun.* 5:5891 doi: 10.1038/ncomms6891 (2014).

## Supplementary Material

Supplementary Information Supplementary Figures 1-14, Supplementary Note 1, Supplementary Methods and Supplementary References.
