# Supplementary material for: Probing short-range protein Brownian motion in the cytoplasm of living cells
Source: Nat Commun. 2014 Dec 23;5:5891. doi: 10.1038/ncomms6891 (PMC4281647; doi:10.1038/ncomms6891)
Supplement: Supplementary Information — Supplementary Figures 1-14, Supplementary Note 1, Supplementary Methods and Supplementary References. [file ncomms6891-s1.pdf]

Supplementary Figures

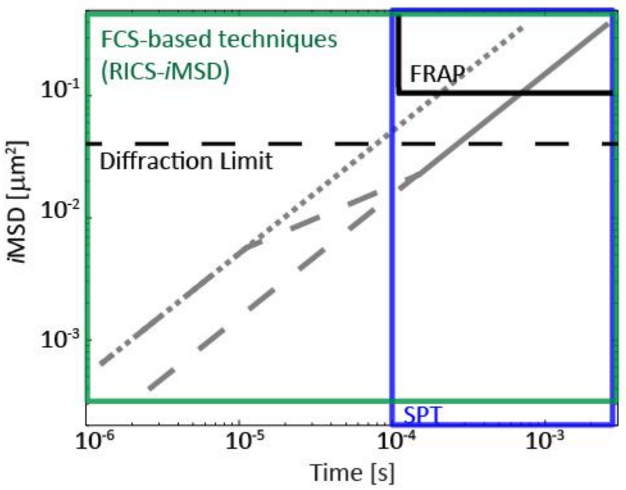

Supplementary Figure 1. Spatiotemporal range available to different techniques. FRAP-based approaches measure the recovery of fluorescence after photobleaching. This ensemble averaging technique, being based on imaging, is limited by a temporal resolution at about 10^{-4} seconds. Moreover, the bleaching spot by a focalized laser is intrinsically limited by diffraction: thus, the spatial resolution of this measurement cannot exceed ~250 nm (black box). SPT is based on particle localization precision. This approach allows gaining a very high spatial resolution and single molecule information that is useful to study heterogeneous environment. However, it usually requires “slow” moving particles and is typically performed in a 2D environment (blue box). Classical FCS measures the average residency time of single molecules in the observed volume: this approach allows a very high temporal resolution but is usually limited by diffraction. However, several variants of this technique has been proposed in the last decades to overcome this limitations including dual-foci FCS, Pair Correlation Functions, STED-FCS, camera-based SPIM FCS, spot-variation-FCS, the Shusterman’s approach, etc. (see main text). We represent the overall potentiality of these approaches by the green box spanning a wide range of both spatial and temporal resolution. Our approach shares the same potentiality, as measure average displacements much smaller than the diffraction limit with a very high temporal resolution. Schematic representation of GFP *i*MSD in solution and in cell reported in the present work (grey lines).

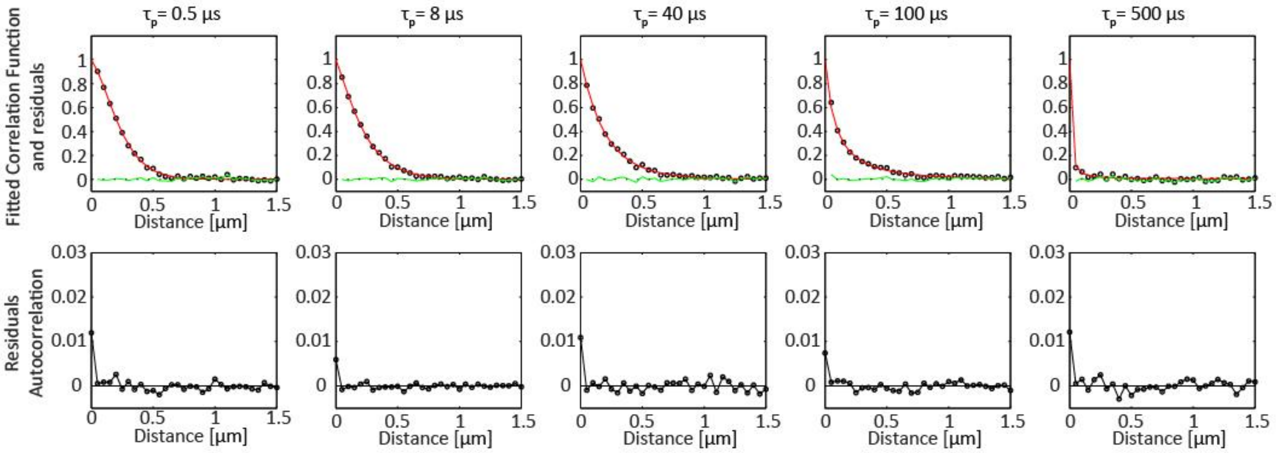

Supplementary Figure 2. Data fitting to Eq. 15, residuals, and residuals autocorrelation for the experimental correlation functions of GFP measured in solution at 37°C (average of N=7 measurements). As expected, the experimental distributions are well described by the Gaussian algorithm.

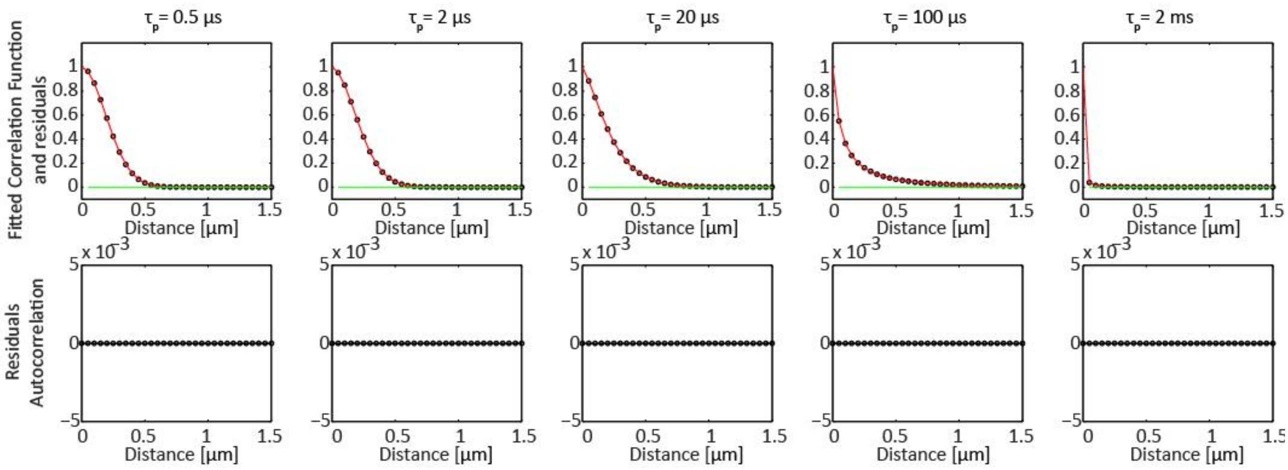

Supplementary Figure 3. A dimensionless particle is left free to diffuse in each direction adding for each time step 't_0' a Gaussian-distributed random number of variance '$2D_0t_0$'. Here we show data fitting to Eq. 15, residuals, and residuals autocorrelation for the simulated correlation functions of particles freely diffusing in 3D. As expected, the obtained correlation functions are well described by the Gaussian algorithm used.

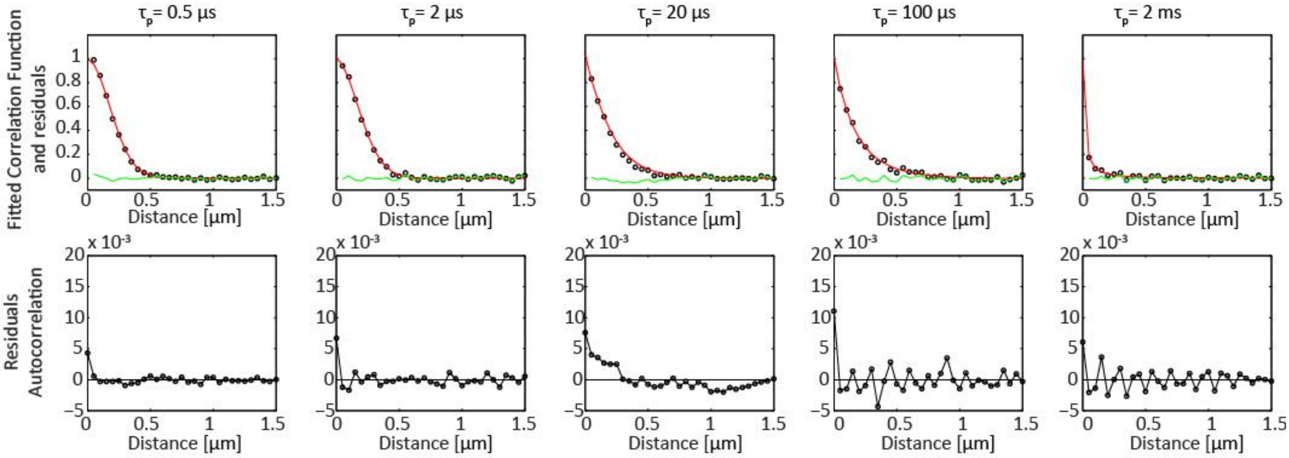

Supplementary Figure 4. Data fitting to Eq. 15, residuals, and residuals autocorrelation for the experimental correlation functions of GFP in live cells at 37°C (average of N=24 cells). Correlation functions are well fitted by Eq. 15 both for short- and long-range particle displacements. Conversely, deviation from the Gaussian approximation is detected at an intermediate scale (20µs pixel dwell-time), corresponding to the anomalous regime.

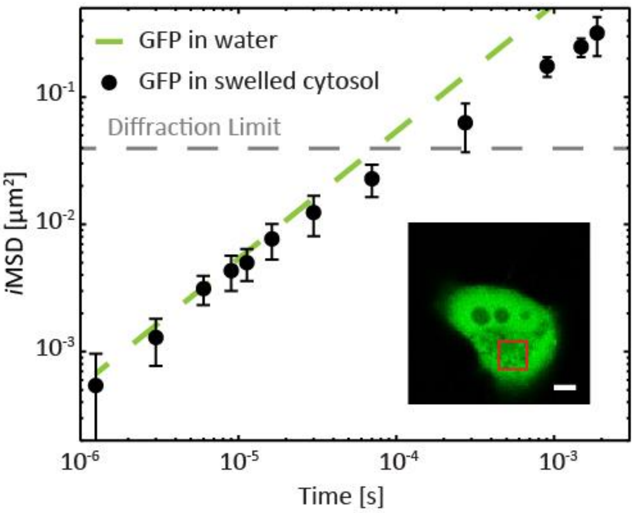

Supplementary Figure 5. GFP motion is affected by hypotonic stress. GFP is transiently transfected into living CHO-K1 cells and analyzed under hypotonic conditions. Arbitrary μm-sized areas in the cell cytoplasm are imaged sequentially at tunable time scales (inset). Exposure of cells to hypotonic conditions produces an expected shift in the position of the crossover between unobstructed and suppressed motion ($t=5 \times 10^{-5}$ s, see Methods). No detectable variation in the short-range diffusivity is observed ($D_0=123\pm4$ μm^2 s^{-1}, 13 cells, N=2 experiments). Scale bar 5 μm. Data are mean values ± s.d.

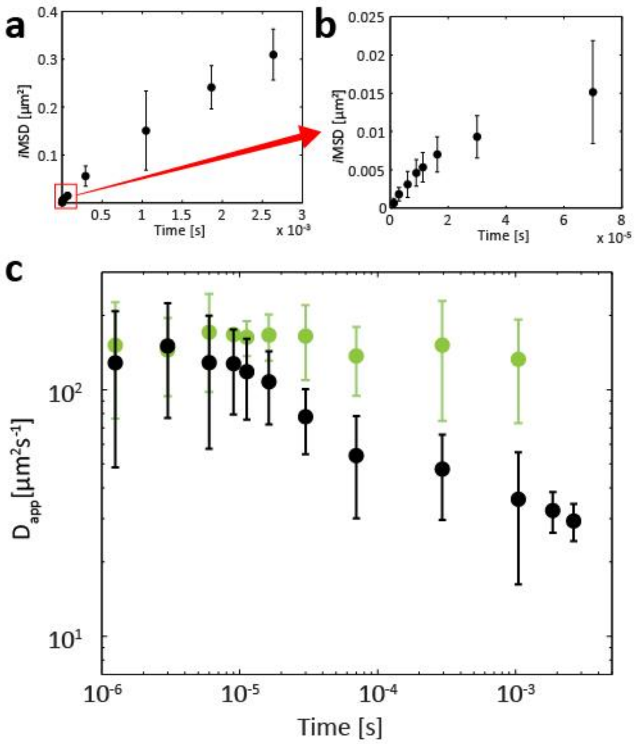

Supplementary Figure 6. Alternative representations of GFP diffusion in the cell cytoplasm. a, double-linear representation of the *i*MSD of GFP over the complete temporal range observed. **b**, selection of the time range below 10^{-4} seconds **c**, from panel (a). **c**, double-logarithmic representation of D_{app} (see Supplementary Methods) as a function of time for both GFP in solution (green dots) and in the cell cytoplasm (black dots). Data are mean values ± s.d.

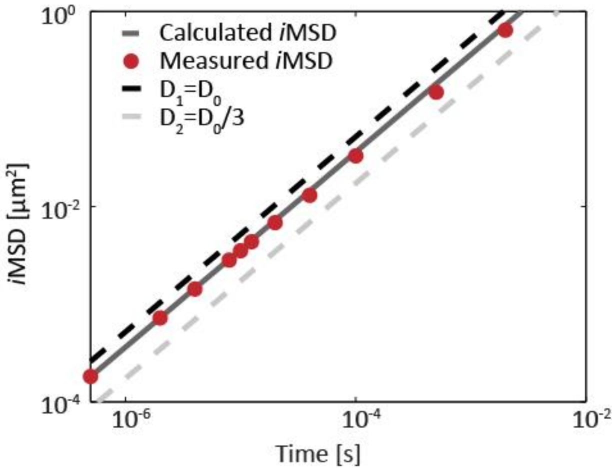

Supplementary Figure 7. *i*MSD for a mixture of two populations of diffusing molecules. When two populations of molecules independently diffuse in 3D with distinct diffusion coefficients (D_1=D_0=130 μm^2 s^{-1}, D_2=D_0/3), the recovered overall *i*MSD (red dots) is linear over the temporal range observed, as expected theoretically (solid grey line). Also, it is not superimposable to any of two isolated components (dashed lines).

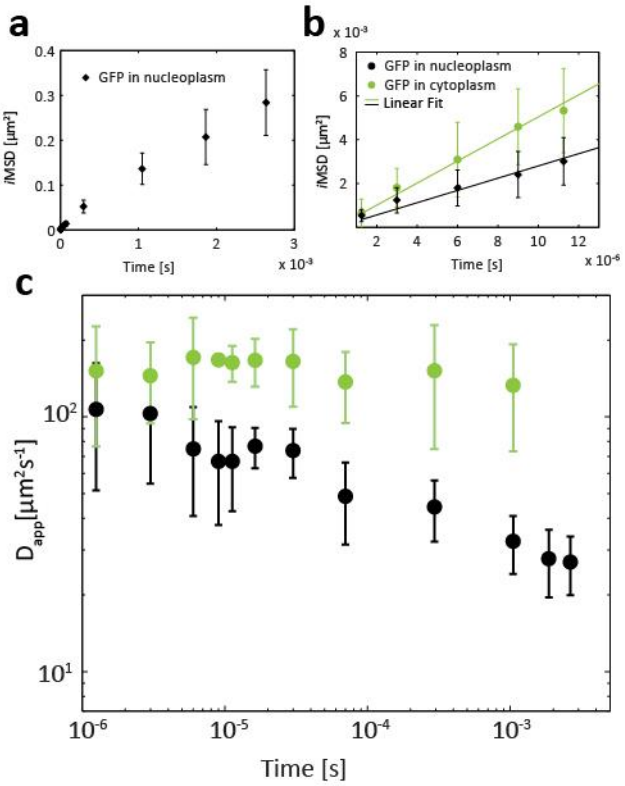

Supplementary Figure 8. Alternative representations of GFP diffusion in the cell nucleus. a, double-linear representation of the *i*MSD of GFP over the complete temporal range observed. **b**, comparison with cytoplasm for a time range below $2x10^{-5}$ seconds. This shows that short-range diffusion of GFP in the nucleoplasm is never unobstructed in the range observed. Linear interpolation of this part of the *i*MSD yields an apparent diffusivity of D_{app}=70±4 μm^2 s^{-1}, that is statistically different from the same quantity calculated for GFP diffusion in the cytoplasm ($p<0.0001$ from t-Student test). **c**, double-logarithmic representation of D_{app} (see Supplementary Methods) as a function of time for both GFP in solution (green dots) and in the cell nucleoplasm (black dots). Data are mean values ± s.d.

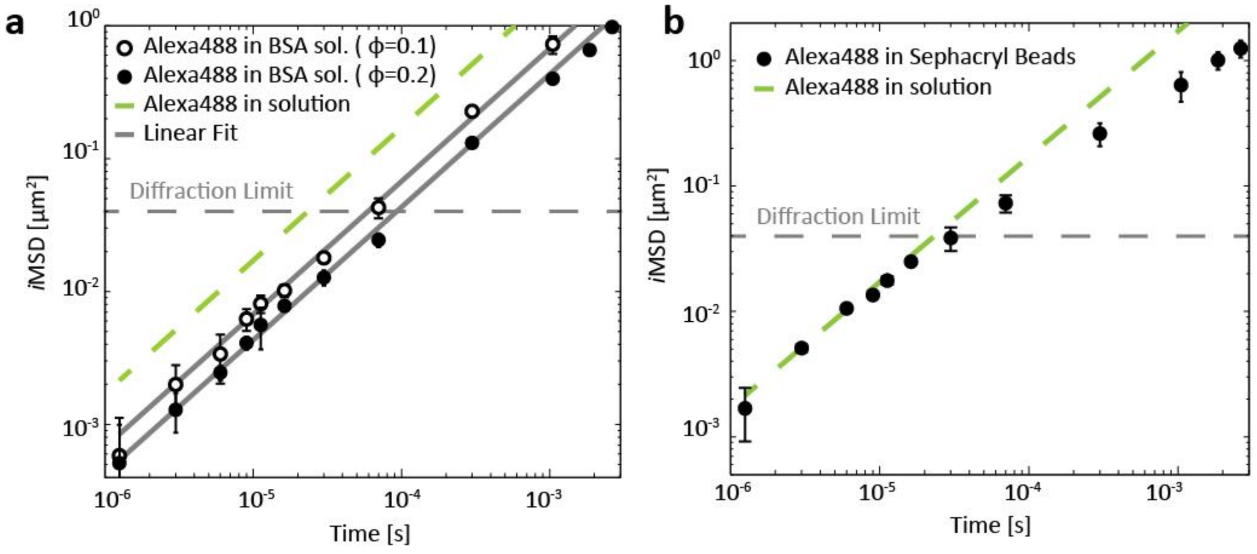

Supplementary Figure 9. *In cuvette* validation of a small tracer dynamics model. a, Experimental *i*MSD of Alexa488 diffusing in BSA solution with different excluded volume fractions (φ=10% and 20%). For both tested φ values, the *i*MSD trend at the different time scales is not distinguishable from free diffusion. At the same time, however, increasing the excluded volume decreases molecular diffusivity (for φ=0.1, D=168±1 $\mu m^2 s^{-1}$and N=7 measurements, for φ=0.2 D=107±1 $\mu m^2 s^{-1}$ and N=7 measurements). **b**, The *i*MSD values of Alexa488 in Sephacryl beads is reported, as average of N=5 beads (black dots), and compared to the *i*MSD calculated in solution outside the beads (dashed green line). The short-range motion (below 2 x 10^{-5} s) is coincident with that in dilute solution, thus it can be well described by free diffusion (D_0=420±5 $\mu m^2 s^{-1}$, χ^2=1.1, $p<0.05$ and N=7 measurements). However, this model does not apply to *i*MSD values above 2 x 10^{-5} s (χ^2>25, p>0.995), analogously to what observed in cells. Data are mean values±s.d.

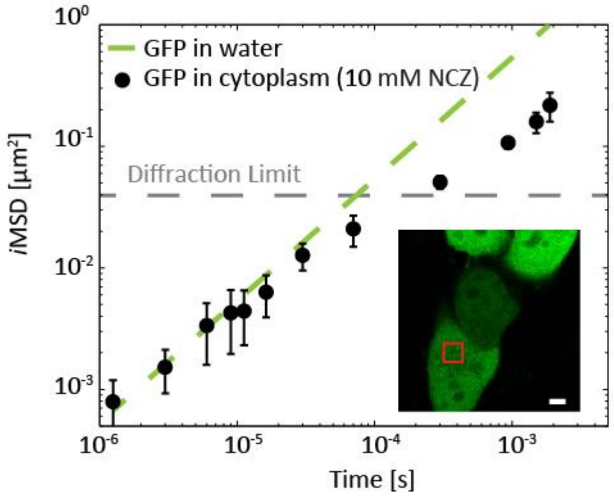

Supplementary Figure 10. *i*MSD of GFP in cytoplasm upon microtubule destabilization. The treatment of cells with 10µM of Nocodazole (NCZ) selectively induces microtubule depolimerization while preserving actin integrity. Interestingly, the obtained *i*MSD is almost coincident with that measured under physiological conditions (see Fig. 3 of the main text), suggesting two important considerations: *i*) the microtubules are not essential to generate GFP overall behavior; *ii*) the increase in soluble protein content in the cell cytoplasm due to microtubule depolimerization is not sufficient to affect GFP short-range motion. Data are mean values±s.d. of 11 cells on N=1 experiment. Scale bar: 5 µm.

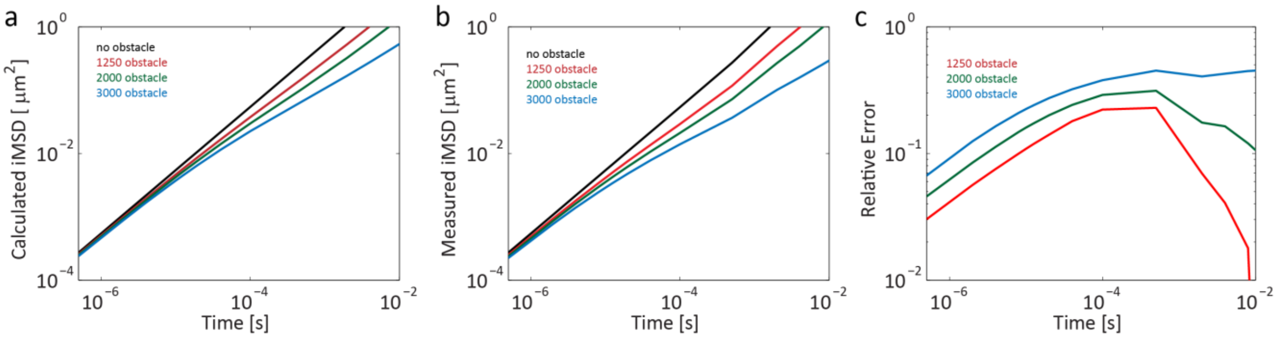

Supplementary Figure 11. Relative error in the measurement of *i*MSD for particles diffusing in heterogeneous environments. a, theoretical *i*MSD calculated from trajectories of particles diffusing in absence or in presence of an increasing number of disk-shaped obstacles (see also Supporting Information for more details). **b**, *i*MSD derived by fitting simulated correlation functions to Eq. 15. **c**, Quantification of the relative error between theoretical and measured *i*MSD quantities. As expected, the maximum relative error occurs when the particle reaches a displacement comparable to the characteristic spatial scale of the obstacles.

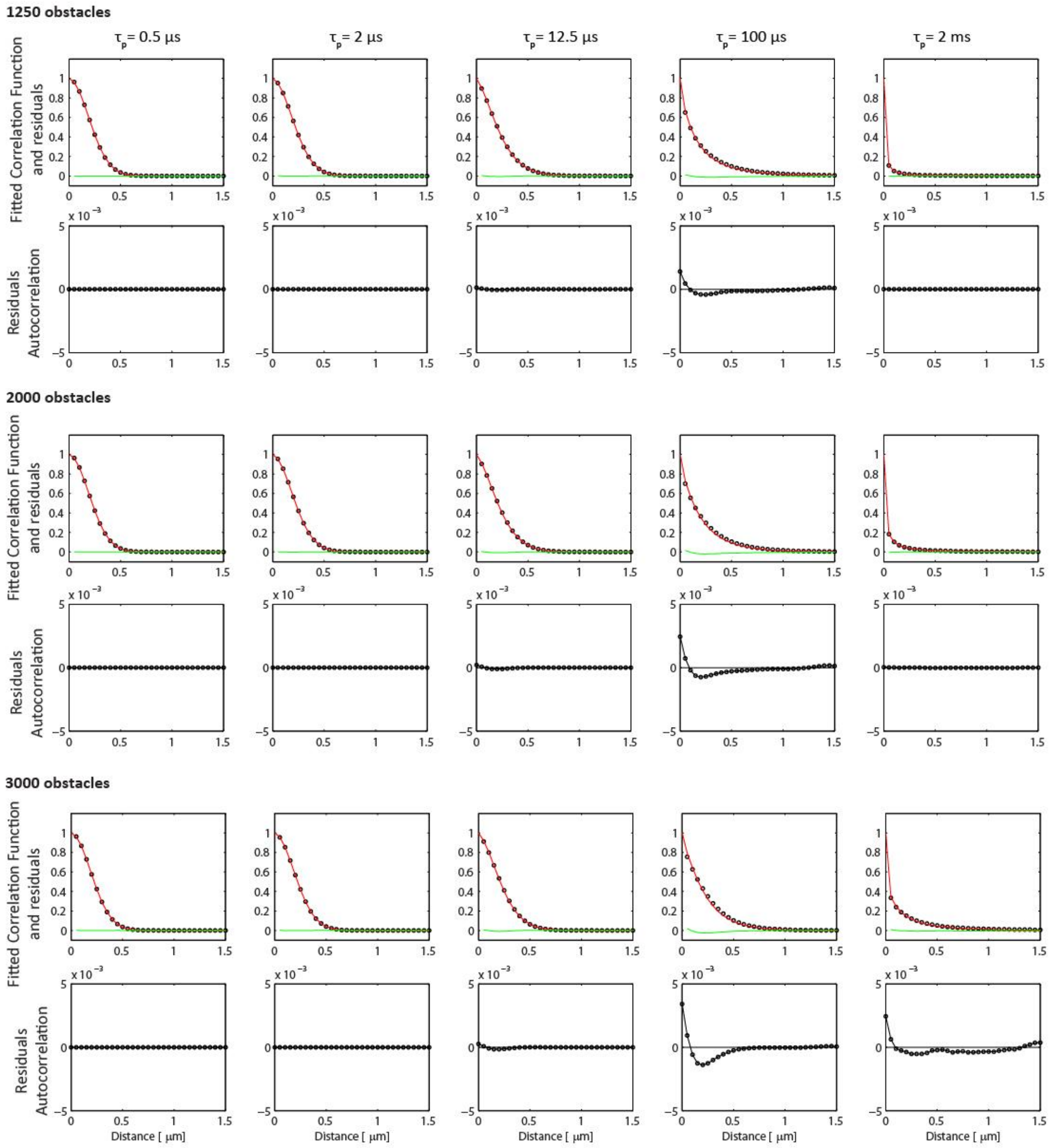

Supplementary Figure 12. A dimensionless particle is left free to diffuse in each direction adding for each time step 't_0' a Gaussian-distributed random number of variance '$2D_0t_0$'. The space is considered with circular boundaries and it is filled with a variable number of randomly distributed overlapping disks (radius 'r' and height 'h') (see Supporting Information for additional details). Here we show data fitting to Eq. 15 for correlation functions calculated from the trajectories of particles. Analogously to what observed in cells, correlation functions are well fitted by Eq. 15 both for short- and long-range particle displacements. Conversely, as expected, deviation from the Gaussian approximation is detected at an intermediate scale, corresponding to the anomalous regime.

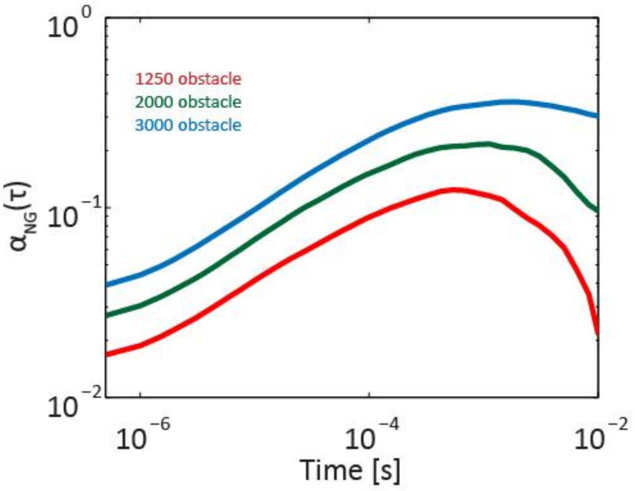

Supplementary Figure 13. Deviation from Gaussian transport at increasing obstacle densities. The ‘non-Gaussian parameter’ $\alpha_{NG}(\tau)$ is calculated from trajectories as described in Supplementary Methods (Eq. 16).

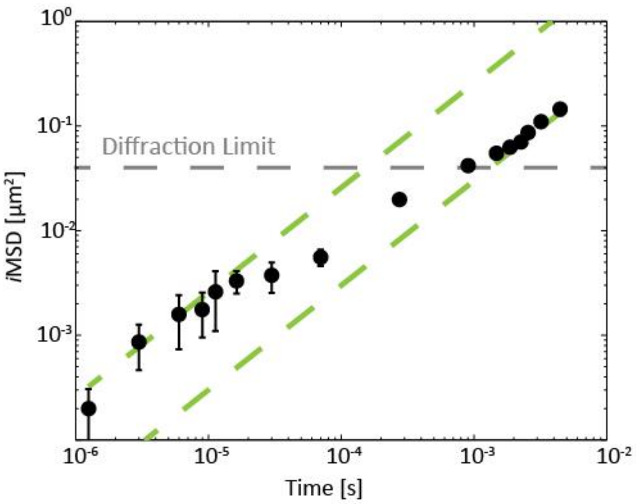

Supplementary Figure 14. *i*MSD analysis of Importin-α-GFP diffusing in the cell cytoplasm. The *i*MSD values at the different time scales are reported in a double-logarithmic representation as average of N=1 experiments, n=9 cells (black dots). D_0 (65±5 µm^2 s^{-1}) and D_{inf} (7.5±2 µm^2 s^{-1}) are estimated by a linear fit of the *i*MSD below 2x10^{-5} s and above 1x10^{-3} s respectively. The green lines represent the expected *i*MSD for normal diffusion with diffusivity D_0 and D_{inf}. Data are mean values ± s.d.

Supplementary Note 1

Comparison with other correlation techniques capable of extracting the MSD. The approach presented here shares many similarities with the multiple scan-speed image correlation spectroscopy (msICS) strategy presented by Groner et al.[1]. However it is worth noting that their aim is completely different: Groner et al. used msICS to build a diffusion map in the cell. Here, instead of using the different pixel dwell/line scan times to generate a temporal autocorrelation function for every pixel shift, we extract the characteristic molecular mean square displacement for each scan speed (*i*MSD). In other words, each scan speed is used as a filter to select the characteristic temporal scale of molecular displacements that significantly contributes to the measured correlation function. Such a conceptual difference leads to different methods of data analysis and data interpretation (see main text). Regarding the comparison with spot-variation FCS[2,3], some connections with our strategy deserve to be highlighted. The two approaches share the concept that selectively observing different (spatial and/or temporal) parts of the whole dynamics can unveil hidden features of particle mobility. Particularly, in spot-variation FCS the size of the spatial filter (the observation volume) is changed and the local dynamics is then obtained by extrapolation. In fact, as elegantly demonstrated by Wawrezinieck et al.[2], local dynamics such as trapping in isolated domains or hopping between adjacent transient confinement zones are very difficult to be distinguished from simple diffusion by means of classical FCS. On the contrary, the possibility to sample different wavenumbers of the scattering function by changing the waist size represents a very successfully strategy. By the present approach, as described above, we are applying temporal filters through which we reconstruct the complete dynamics. A different successful approach to exploit the huge amount of information contained in the FCS correlation function has been presented by Shusterman et al.[4]. The authors technically use the equivalent of Eq. 11 of the present work to directly extract the MSD of a particle diffusing in 3D. Despite the analogy, the obtained information can be substantially different in the two cases. In fact, the MSD measured by Shusterman et al. represents local information related to the spatial scale of the observation volume

waist. Although in a homogenous environment (e.g. dilute solution) this local information (averaged in time during the acquisition) can be considered as representative of the whole sample, the same paradigm does not apply in cells where structures such as membranes, cytoskeleton, vesicles and organelles may regulate molecular diffusion in a different way depending on the particular position in space. Thus we decided to follow a spatio-temporal approach able to average together all the possible positions in space. In fact, with our approach the particle displacement is measured as averaged on a wide cell portion of several microns while each point of the *i*MSD plot represents an independent measurement (although performed on the same portion of the cell).

Supplementary Methods

From RICS in tunable time scales to *i*MSD: theory. The theoretical foundation of RICS for the case of normal Brownian diffusion has been published previously[5]. Here we shall consider a general case of transport in order to highlight the potential of our approach to address molecular dynamics with very high spatio-temporal resolution. In particular, we will follow the approach already presented by Shusterman et al.[4] and further generalized by Hofling et al.[6] for FCS. We will tackle the case of mono-dimensional scanning in the *x*-direction. In particular, we will derive the equations in a reference system with the origin is set at the center of the observation volume. We define $\Delta\mathbf{R}(\tau)$ as a random variable that represents the displacement of the molecule during the time τ from its position at time 0; thus, we can define $\Delta\mathbf{R}^*(\tau) = \Delta\mathbf{R}(\tau) + \mathbf{v}\tau$ as the displacement in the chosen reference system, where $\mathbf{v}$ represents the scan speed. Now we define the measured fluorescence as:

$$F(\tau) = \varepsilon \int d\mathbf{r} W(\mathbf{r}) c(\mathbf{r},\tau) \tag{1}$$

where $W(\mathbf{r})$ represents the observation volume, $c(\mathbf{r},\tau)$ the concentration of the fluorescent molecule in time and space (this term contains the physics of the transport process) and ε the total efficiency of the acquisition. Furthermore, we define the correlation function as:

$$G(\tau) = \frac{\langle \delta F(\tau) \delta F(0) \rangle}{\langle F \rangle^2} \tag{2}$$

where $\langle \cdots \rangle$ represents the average and $\delta F(\tau) = F(\tau) - \langle F \rangle$. The equation for $G(\tau)$ can be obtained by integration in the reciprocal space '$\mathbf{k}$', defining the intermediate scattering function as:

$$S(\mathbf{k},\tau) := \frac{1}{\langle c \rangle} \int d\mathbf{r} e^{-i\mathbf{kr}} \langle \delta c(\mathbf{r},\tau) \delta c(\mathbf{0},0) \rangle \tag{3}$$

and obtaining:

$$G(\tau) = \frac{1}{N}\frac{\int d^d k |W(\mathbf{k})|^2 S(\mathbf{k},\tau)}{\int d^d k |W(\mathbf{k})|^2} \quad (4),$$

where $W(\mathbf{k})$ represents the Fourier transform of $W(\mathbf{r})$ and N the average number of molecules in the observation volume. Thanks to the dilute concentration of the label, the intermediate scattering function can be approximated as the characteristic function of single molecule displacements, according to:

$$S(\mathbf{k},\tau) \approx P(\mathbf{k},\tau) = \left\langle e^{-i\mathbf{k}\Delta\mathbf{R}^*(\tau)} \right\rangle \quad (5),$$

Now we can introduce the commonly used Gaussian profile approximation of the observation volume as:

$$W(\mathbf{r}) = W(0)\exp\left(-2\frac{x^2+y^2}{\omega^2} - 2\eta^{-2}\frac{z^2}{\omega^2}\right) \quad (6),$$

where $\eta^2 = \frac{z_0^2}{\omega^2}$ represents the anisotropy ratio for the illumination volume (z_0^2 is the waist size in the axial dimension). Thus, the correlation function can be expressed in its general form as:

$$G(\tau;\omega,v) = \frac{1}{N}\left\langle \exp\left(-\frac{(\Delta X(\tau)+v\tau)^2}{\omega^2} - \frac{\Delta Y(\tau)^2}{\omega^2} - \frac{\eta^{-2}\Delta Z(\tau)^2}{\omega^2}\right)\right\rangle \quad (7),$$

where $\Delta X(\tau)$, $\Delta Y(\tau)$ and $\Delta Z(\tau)$ represent the molecular displacements in the 3 axial directions. Please note that this definition takes into account the Gaussian approximation of the PSF, but no hypotheses are made on the nature of the molecular transport. Starting from this equation, it is easy to verify that, if we assume that the scanning speed approaches to infinity, the imaging contribution ($\exp\left(-\frac{v^2\tau^2}{\omega^2}\right)$) will dominate the correlation function. Under this condition we can consider

$2\frac{\Delta X(\tau)}{v\tau} + \frac{\Delta X(\tau)^2 + \Delta Y(\tau)^2 + \eta^{-2}\Delta Z(\tau)^2}{v^2\tau^2} << 1$ obtaining:

$$G(\tau;\omega,v\to\infty)=\frac{1}{N}\left\langle \exp\left[-\frac{v^2\tau^2}{\omega^2}\right]\right\rangle=\frac{1}{N}\exp\left[-\frac{v^2\tau^2}{\omega^2}\right] \qquad (8)$$

that represents, as expected, the well-known spatial correlation function[7]. Moreover, if we consider the case of $v\to 0$, the de-correlating contribution due to the molecular movement ($\exp\left(-\frac{\Delta X(\tau)^2}{\omega^2}-\frac{\Delta Y(\tau)^2}{\omega^2}-\frac{\eta^{-2}\Delta Z(\tau)^2}{\omega^2}\right)$) will dominate and Eq. 7 reduces to the FCS case.

Particularly, if the central limit theorem holds, we can consider a Gaussian transport by defining the van Hove self-correlation function as:

$$P_{\text{gauss}}(\mathbf{r},\xi)=\frac{1}{\left[2\pi\delta r^2(\xi)/d\right]^{d/2}}\exp\left(-\frac{r^2}{2\delta r^2(\xi)/d}\right) \qquad (9),$$

where $\delta r^2(\tau)$ represents the Mean Square Displacement. Following the approach we presented in a previous report[8], we define the *i*MSD as the width of the van Hove self-correlation function, and consequently:

$$iMSD(\tau)\overset{def}{=}2\delta r^2(\tau)/d \qquad (10),$$

In light of Eqs. 9-10, we can obtain:

$$G_{\text{Gauss}}(\tau;\omega,v\to 0)=\frac{1}{N}\left[1+\frac{2\delta r^2(\tau)}{d\omega^2}\right]^{-1}\left[1+\frac{2\delta r^2(\tau)}{dz_0^2}\right]^{-1/2}=\frac{1}{N}\left[1+\frac{iMSD(\tau)}{\omega^2}\right]^{-1}\left[1+\frac{iMSD(\tau)}{z_0^2}\right]^{-1/2} \qquad (11)$$

that is homologous to the equation for the single-point FCS case[4,6]. Finally, we can consider the case in which the scanning speed and the molecular movement take place on a similar spatiotemporal scale. In this intermediate situation, we have a significant contribution from the scanning component ($\exp\left(-\frac{2\Delta X(\tau)v\tau}{\omega^2}\right)$) of Eq. 7. At this point, considering valid Eq. 9, we can calculate the RICS correlation function in the case of Gaussian transport as:

$$G_{\mathrm{Gauss}}(\tau;\omega,v)=\frac{1}{N}\left[1+\frac{iMSD(\tau)}{\omega^2}\right]^{-1}\left[1+\frac{iMSD(\tau)}{z_0^2}\right]^{-1/2}\exp\left(-\frac{v^2\tau^2}{iMSD(\tau)+\omega^2}\right) \quad (12).$$

If we consider the case of simple Brownian diffusion, where:

$$iMSD(\tau)=4D\tau \quad (13),$$

then Eq.12 can be reduced to a mono-dimensional analogous of the well-known RICS equation[5].

From RICS in tunable time scales to *i*MSD: experimental realization.

In classical RICS, in order to describe the shape of the correlation function in space, we need to set the distance between adjacent pixels lower than the instrumental waist. However, the maximum scan speed achievable is limited by the pixel size. In order to maximize the speed, we fix the pixel size to 50 nm while different scan speeds are obtained increasing the pixel dwell-time. Also, longer time delays have been obtained by exploiting the direction perpendicular to scanning, where the time lag between adjacent pixels is represented by the line time. In practice, several sequential images of the same region are acquired at the selected scan speed and the average correlation functions both in the scanning and in the perpendicular direction are calculated. Each correlation function is characterized by the corresponding time delay, from $5{*}10^{-7}$s to 10^{-2} s (and by a scanning speed that consequently spans from 10^5 to 5 μm s^{-1}). Ideally, the RICS functions obtained in this way could be directly inverted by Eq. 12, thus obtaining the $iMSD(\tau)$ parameter. Unfortunately, RICS correlation functions obtained in live cell are usually noisy, particularly at low scan-speed (see Supplementary Figure 4, for pixel dwell times ≥100 μs). In this context, in order to obtain a more robust strategy that can be easily and extensively applicable in a live cell, we take advantage of the spatiotemporal sampling of the molecular displacement using different scanning speeds. Particularly, at each scanning speed selected, only the molecular displacements at the timescale comparable to the pixel dwell-time (τ_{p}) significantly contribute to determine the shape of the correlation function (with no contribution arising from displacements that take place on a time scale

significantly greater or smaller than τ_{p}). In other words, the scanning speed acts as a temporal filter selecting the scale of the intermediate scattering function that significantly contributes to the measured correlation function. Following this reasoning, we define for each scanning speed:

$$iMSD(\tau) \approx iMSD(\tau_{\mathrm{p}})\xi \qquad (14),$$

which allows in turn to recast Eq. 12 into:

$$G(\xi) \approx \frac{1}{N}\frac{1}{\left(\omega_0^2 + iMSD(\tau_{\mathrm{p}})\xi\right)}\frac{1}{\sqrt{\omega_0^2\rho^2 + iMSD(\tau_{\mathrm{p}})\xi}}\exp\left(-\frac{(\delta r\xi)^2}{\omega_0^2 + iMSD(\tau_{\mathrm{p}})\xi}\right) \qquad (15)$$

where $\xi = \dfrac{v\tau}{\delta r_{\mathrm{p}}}$ and δr_{p} represents the pixel size. This approximated form of Eq. 12 allows a fast and robust point-by-point estimation of the $iMSD(\tau)$ in live cells, despite the quite low signal-to-noise ratio typical of these measurements, particularly at low scan speeds. Finally, the measured $iMSD(\tau_{\mathrm{p}})\xi$ can be used to estimate the apparent diffusion coefficient (D_{app}) at the corresponding time scale as $D_{\mathrm{app}} = \dfrac{iMSD(\tau_{\mathrm{p}})}{4\tau_{\mathrm{p}}}$.

From RICS in tunable time scales to *i*MSD: data interpretation.

The approximations described above, that are obviously valid for the Brownian case, cannot be taken for granted in a biological sample where the dynamics is usually suppressed (i.e. the ideal solution condition does not apply). In the present work we focus on the study of nanoscopic inert molecules, particularly GFPs. Under this condition we consider all the other actors in solution as obstacles to GFP movement. Two main classes of obstacles can be considered responsible for the observed suppressed translational motion of GFP in the live cell environment: *i*) molecules homogeneously diffusing and colliding; *ii*) immobile and spatially-organized intracellular structures (heterogeneous mixture). Both *in silico*[9] and *in vitro*[10] experiments demonstrated that, in the former case, translational diffusion is already suppressed at the molecular scale (<10 nm). Thus, we expect

that Eq. 15 represents a good approximation for this case. We believe that a direct proof of this assumption is provided by the results obtained for GFP diffusion in crowded solution of albumin (Fig. 4). The spatial heterogeneity in biological environments is mainly attributed to the presence of solid structures (e.g. ER membranes, Golgi apparatus, mitochondria, vesicles, cytoskeleton, etc.) suspended in a liquid phase. This system is usually simulated as a pattern of impenetrable immobile obstacles, and theoretically interpreted according to the 'Lorentz' model, that accounts for the movement of molecules in a heterogeneous environment where excluded volume effects dominate[11]. Recent *in silico* results by Hofling et al.[12,13] show that the distribution of spatial displacements of particles in a Lorentz model significantly deviates from a Brownian behavior only for average displacements that are comparable to the characteristic distance between the obstacles. In this regime a significant deviation from Gaussian transport is observed. Instead, for average displacements significantly smaller or greater than this characteristic threshold, particle movement is not distinguishable from a Brownian motion. To quantitatively assess the consistency of our approximations in this case, we set a series of simulations following a Monte Carlo approach. Particularly, a dimensionless particle is left free to diffuse in each direction adding for each time step t_0=50 ns a Gaussian-distributed random number of variance $2D_0t_0$ (D_0=130 μm^2 s^{-1}). The space was considered with circular boundaries and it was filled of a variable number of randomly distributed overlapping disks (radius r=300 nm and height h=100 nm) following the approach previously introduced by Novak et al.[14] to reproduce the obstructed environment imposed by cellular structures (e.g. membrane layers) in the cytoplasm. The obstructed motion was reproduced as follows: when the particle attempts to enter into an obstacle, it is stopped and a new step is run. In order to characterize the simulated dynamics, the particle trajectories were used to calculate the *i*MSD and the 'non-Gaussian parameter' ($\alpha_{NG}(\tau)$), defined as reported by Hofling and Franosh[11]:

$$\alpha_{\mathrm{NG}}(\tau)=\frac{d}{d+2}\frac{\delta r^4(\tau)}{\left[\delta r^2(\tau)\right]^2}-1 \qquad (16)$$

where $\delta r^4(\tau)$ is the mean quartic displacement. This is a simple dimensionless indicator for transport beyond the Gaussian approximation. Such phenomena are expected to play a role, for instance, in the case of particle diffusion within obstructed environments[12]. The correlation functions obtained for all the selected scanning speeds (using Eq. 7) are then fitted to Eq. 15 in order to measure the *i*MSD by our approach. Different obstacles concentration were characterized by the ratio between the imposed diffusivity (D_0) and the apparent diffusivity ($D_{\text{app}} = \frac{iMSD(t)}{4t}$) measured at t=10 milliseconds. Supplementary Figure 12 shows the agreement between the simulated correlation function and Eq. 15. In particular, for all simulated conditions Eq. 15 well describes the correlation function for a wide range of pixel dwell times. At slower scan speeds, significant deviation from Gaussian transport can be observed only for the highest obstacle density simulated (that reduces diffusivity of a factor of 10, bottom-right corner in Supplementary Figure 12). Also, please note that such deviation is transient as it disappears for long-range particle displacements. Supplementary Figure 11 compares the measured *i*MSD with the expected one. Notably, the measured *i*MSD correctly unveils the deviation from Brownian dynamics with only a slight underestimation of the expected values. Moreover, particularly for the reduction in diffusivity of a factor of 3, as estimated in live cell by us and others (see main text), this deviation is at maximum 20%, lower than the experimental error. Based on these simulations we believe that our strategy can probe the correct dynamics of GFP in live cells.

Supplementary References

1 Groner, N., Capoulade, J., Cremer, C. & Wachsmuth, M. Measuring and imaging diffusion with multiple scan speed image correlation spectroscopy. *Opt Express* **18**, 21225-21237 (2010).

2 Wawrezinieck, L., Rigneault, H., Marguet, D. & Lenne, P. F. Fluorescence correlation spectroscopy diffusion laws to probe the submicron cell membrane organization. *Biophys. J.* **89**, 4029-4042, doi:10.1529/biophysj.105.067959 (2005).

3 Lenne, P. F. *et al.* Dynamic molecular confinement in the plasma membrane by microdomains and the cytoskeleton meshwork. *EMBO J.* **25**, 3245-3256, doi:10.1038/sj.emboj.7601214 (2006).

4 Shusterman, R., Alon, S., Gavrinyov, T. & Krichevsky, O. Monomer dynamics in double- and single-stranded DNA polymers. *Phys Rev Lett* **92**, 048303 (2004).

5 Digman, M. A. *et al.* Measuring fast dynamics in solutions and cells with a laser scanning microscope. *Biophys. J.* **89**, 1317-1327, doi:10.1529/biophysj.105.062836 (2005).

6 Hofling, F., Bamberg, K. U. & Franosch, T. Anomalous transport resolved in space and time by fluorescence correlation spectroscopy. *Soft Matter* **7**, 1358-1363, doi:Doi 10.1039/C0sm00718h (2011).

7 Petersen, N. O., Hoddelius, P. L., Wiseman, P. W., Seger, O. & Magnusson, K. E. Quantitation of membrane receptor distributions by image correlation spectroscopy: concept and application. *Biophysical journal* **65**, 1135-1146, doi:10.1016/s0006-3495(93)81173-1 (1993).

8 Di Rienzo, C., Gratton, E., Beltram, F. & Cardarelli, F. Fast spatiotemporal correlation spectroscopy to determine protein lateral diffusion laws in live cell membranes. *Proceedings of the National Academy of Sciences of the United States of America* **110**, 12307-12312, doi:10.1073/pnas.1222097110 (2013).

9 Trovato, F., Nifosì, R., Di Fenza, A. & Tozzini, V. A Minimalist Model of Protein Diffusion and Interactions: The Green Fluorescent Protein within the Cytoplasm. *Macromolecules* **46**, 8311-8322 (2013).

10 Roosen-Runge, F. *et al.* Protein self-diffusion in crowded solutions. *Proc Natl Acad Sci U S A* **108**, 11815-11820 (2011).

11 Hofling, F. & Franosch, T. Anomalous transport in the crowded world of biological cells. *Rep Prog Phys* **76**, 046602 (2013).

12 Hofling, F., Franosch, T. & Frey, E. Localization transition of the three-dimensional lorentz model and continuum percolation. *Phys Rev Lett* **96**, 165901 (2006).

13 Hofling, F., Munk, T., Frey, E. & Franosch, T. Critical dynamics of ballistic and Brownian particles in a heterogeneous environment. *J Chem Phys* **128**, 164517 (2008).

14 Novak, I. L., Kraikivski, P. & Slepchenko, B. M. Diffusion in cytoplasm: effects of excluded volume due to internal membranes and cytoskeletal structures. *Biophys J* **97**, 758-767 (2009).
